# Potential Method to Distinguish Copper Molten Marks Using Boundary and Grain Characteristics

**DOI:** 10.3390/ma15134534

**Published:** 2022-06-28

**Authors:** Jinyoung Park, Joo-Hee Kang, Hyo-Sun Jang, Young Ho Ko, Sun Bae Bang

**Affiliations:** 1 Korea Electrical Safety Corporation Research Institute, 111, Anjeon-ro, Iseo-myeon, Wanju-gun 55365, Jeollabuk-do, Korea; jyp82@kesco.or.kr (J.P.); bsb1586@kesco.or.kr (S.B.B.); 2 Korea Institute of Materials Science, 797, Changwon-daero, Seongsan-gu, Changwon-si 51508, Gyeongsangnam-do, Korea; hsjang@kims.re.kr; 3 Jeonbuk National University, 567, Baekje-daero, Deokjin-gu, Jeonju-si 54896, Jeollabuk-do, Korea; koyh@jbnu.ac.kr

**Keywords:** molten mark, primary-arc bead, secondary-arc bead, electron-backscatter diffraction, Σ3 boundary, grain size, machine learning

## Abstract

The microstructure of molten marks changes according to ambient temperatures, when a short circuit occurs. Investigation of microstructural changes is important for understanding the properties of copper and examining the cause of a fire. In this study, the boundary characteristics and grain-size distribution of molten marks—primary-arc beads (PABs), which short-circuited at room temperature (25 °C), and secondary-arc beads (SABs), which short-circuited at high temperatures (600 °C, 900 °C)—were compared using electron backscatter diffraction. The distribution of Σ3 boundaries was compared, and it was found that SABs have a higher fraction of Σ3 boundaries than PABs. Moreover, it was confirmed that the ratio of maximum grain size (area) to the total area of the molten mark in SABs is larger than that in PABs. Thus, reliable discriminant factors were suggested, such as the fraction of Σ3 boundaries and normalized maximum grain size, which can distinguish PABs and SABs. The four discriminant factors, such as the (001)//LD, GAR, fraction of Σ3 boundaries, and fraction of maximum grain size to the total molten-mark area, were verified using the machine learning of t-SNE and Pearson correlation analyses.

## 1. Introduction

The short-circuit arc in electrical wires, caused by defective and worn insulation, accounts for 30% of the leading causes of electric fires in residential buildings [1]. However, as 51% of fires caused by electrical problems at home were an unclassified electrical failure and malfunction [2], it is expected that more electric fires related to electrical wires may have occurred. One of the reasons for the undetermined cause is that it is unclear whether the molten marks identified on the wires are the cause or the result of the fire. Therefore, if the cause of the formation of molten marks is clearly known, fires of undetermined causes can be reduced.

A molten mark, caused by electrical or thermal heat, refers to a bead solidifying after the wire melts. Molten marks on wires often found at fire sites can be roughly characterized into three types [3]: (i) primary-arc beads (PABs), which are produced by arcs caused by a short circuit after damage by insulation deterioration or an external physical force; (ii) secondary-arc beads (SABs), which are molten marks formed by short circuit arc when the insulation of the wire melts owing to an external flame; and (iii) globules, which refer to the molten marks that are solidified after the copper wire melts under an external flame in a nonenergized state. 

Since fire investigations are performed by examining the presence or absence, shape, and appearance of molten marks, distinguishing the type of the molten marks is essential in identifying the location and causes of fires. Discriminating between short-circuit molten marks (PABs, SABs) and globules is possible with the naked eye [4]. However, accurately discriminating PABs and SABs is challenging. For this reason, various criteria have been suggested to distinguish PABs from SABs, including the number or size of voids in the molten marks [5,6,7,8]; the spacing between branches of dendritic structure [9]; the existence of amorphous or graphitic carbon in the molten marks [10]; and the oxygen-, carbon-, and chlorine-concentration distribution of the molten marks [11,12]. Nevertheless, many proposed criteria were difficult to apply for distinguishing between PABs and SABs, due to the lack of quantification and verification [13].

Accordingly, identifying quantitative methods for discriminating PABs and SABs is essential for fire investigations. Thus, this study aimed to identify the objective factors that can distinguish the types of molten marks using electron-backscatter diffraction (EBSD). EBSD can produce reliable results because it quantitatively analyzes the microstructure and crystal orientations of molten marks.

In a previous study, Park et al. [14] suggested the (001) crystal-direction fraction perpendicular to the demarcation line and the grain-aspect ratio as factors to distinguish PABs and SABs. However, as the number of discriminant factors increases, the probability of discrimination (reliability) also increases because the molten marks can be analyzed through various aspects. This study investigated new discriminant factors by assessing microstructural characteristics such as misorientation and grain size. Various arc-bead-formation conditions were considered, including copper wires shorted at 25 °C, 600 °C, and 900 °C to produce molten marks. 

## 2. Materials and Methods

### 2.1. Materials and Apparatus

**Figure 1:**
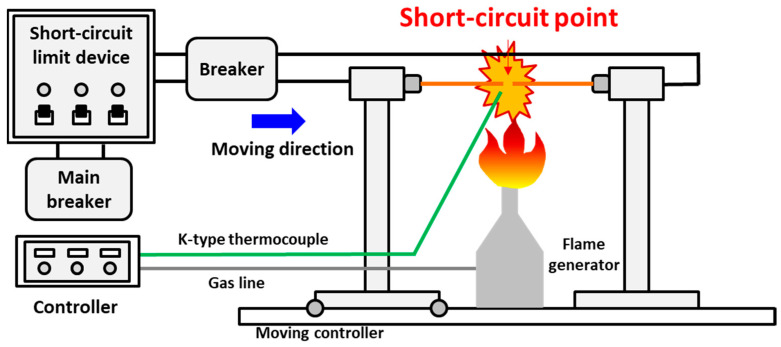
Schematic illustration of the short-circuit generator.

Figure 1 shows the experimental setup for preparing PABs and SABs. The experimental procedure was described in reference [14]. The wires used in this experiment are electrolytic tough-pitch (ETP) copper, which is at least 99.90% pure and has an electrical conductivity of at least 101% of the International Annealed Copper Standard (IACS)’s minimum. Bare copper wires (diameter: 1.6 mm) were fixed to each holder, and the left wire was moved toward the right wire using a control device to induce a short circuit between the two wires. This method was adopted to maintain a constant exposure time to the external flame and allow data acquisition under fixed conditions. A gas burner was installed below the short-circuit point to control the ambient temperature during the short circuit. The flame temperature of the gas burner can be controlled up to 1300 °C. A K-type thermocouple (temperature range: −230 °C to 1250 °C, limits of error: ±2.2 °C) was installed to measure the temperature at a distance of 2 mm from the short-circuit point in the horizontal direction. The temperature difference between short-circuit point and ambient air was 22 °C when the target temperature of 900 °C was stabilized [14]. The ambient temperature can be regarded as the temperature of the copper conductor.

The short-circuit current was limited to 500 A [15] using a short-circuit-limiting device manufactured based on the UL 1699 standards (UL Standards, Northbrook, IL, USA) to reduce the effect of the short-circuit’s current magnification on the molten mark’s shape.

### 2.2. Fabrication of Molten Marks

PAB-25 was produced by shorting at an ambient temperature of 25 °C, assuming that insulation diminishes and a direct shorting of metallic conductors occurs. Meanwhile, PAB-600 was created by reheating the PAB-25 to 600 °C for 5 min, assuming a fire occurred after the short circuit.

The temperature at which the fire spreads and flashover occurs was approximately 600 °C [4]. At this stage, the probability of a wire short-circuit increases. In addition, Weinschenk et al. [16] reported that within 2 min of applying a flame, the temperature started to rise, and over-current faults or grounding faults occurred; the ambient temperature at this time was approximately 900 °C. Based on these two reports, the temperature of the copper wires was maintained at 600 °C and 900 °C for 2 min, and SAB-600 and SAB-900 were then, respectively, produced by inducing a short circuit. After that, the temperature was maintained for 5 min. Experimental conditions for the fabrication of the molten marks are presented in Table 1.

### 2.3. Specimen-Measurement Method and Analysis Tool

A field emission-scanning electron microscope (FE-SEM; Hitachi SU-70, Japan) equipped with EBSD (EDAX Hikari, Pleasanton, CA, USA) was used to obtain the orientation data of the molten marks. The accelerating voltage used was 15 kV, and the probe current was 15 nA. The specimens were mapped to a hexagonal grid at a step size of 4 μm. For EBSD observations, the produced molten marks were mounted with conductive resin and mechanically polished with SiC paper, a diamond suspension, and colloidal silica suspension [17,18].

The post-processing for quantitative microstructure characterization was performed using the orientation imaging microscopy (OIM)-analysis software version 8.6 (EDAX-TSL, Pleasanton, CA, USA). The grain boundary angle (grain tolerance angle) was set to 5°, and the orientation reliability (confidence index, CI) was set to 0.2 or more. Grain CI standardization and neighbor-orientation correlation (level 4) were used to clean up the data. The misorientation angle (*θ*) and coincidence-site lattice (CSL) boundaries for the PABs and SABs were compared to analyze misorientation differences.

## 3. Results and Discussion

### 3.1. Decision of the Demarcation Line

As the molten mark formed on the copper wire by the short-circuit energy solidifies rapidly, a demarcation line is formed between the melted and nonmelted parts [19,20]. To distinguish molten marks using quantitative methods such as boundary characteristics, it is necessary to define a demarcation line between the melted and nonmelted parts. However, a clear criterion to define the demarcation line is not available. In this study, a criterion was suggested using the unique characteristics of the columnar structure in the melted part. The fine and equiaxed grains are developed near the demarcation line because of the rapid cooling and solidification. However, the small number of fine grains formed, even in the melted part, are excluded for clear discrimination. 

Grain aspect ratio (GAR) was used as a parameter to decide the columnar structure. It is the length of the minor axis divided by the length of the major axis. The closer the GAR is to 0, the more elliptical it is, and the closer it is to 1, the more circular it is. 

**Figure 2:**
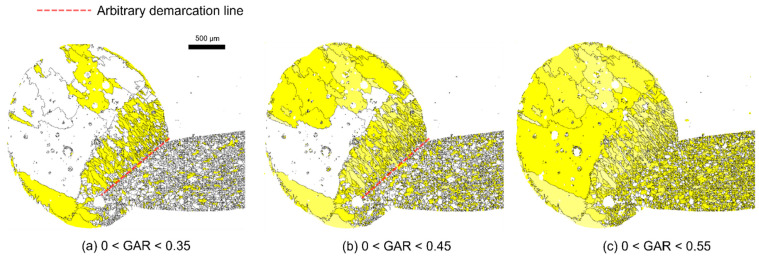
Changes in columnar-structure region (yellow color) according to grain aspect ratio (GAR) values. Red dotted line is the arbitrary demarcation line. (**a**) 0 < GAR < 0.35, (**b**) 0 < GAR < 0.45, (**c**) 0 < GAR < 0.55.

**Figure 3:**
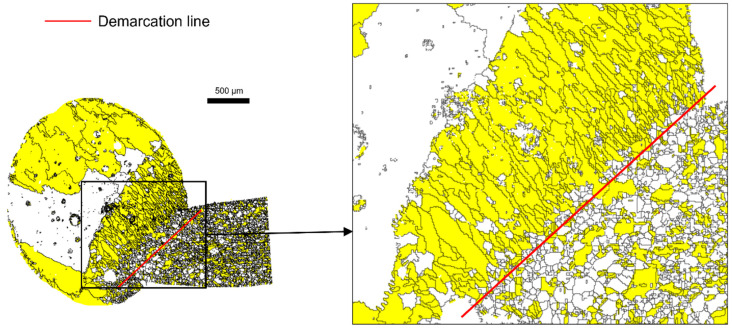
Set demarcation line of the molten mark: the region with grain aspect ratio (GAR) < 0.45 (yellow color) and demarcation line (red line).

As shown in Figure 2, columnar grains (yellow area) changes were observed as the GAR was changed from 0 to a specific value. The columnar grains were developed mainly in the molten parts. Using these microstructure characteristics, the melted and nonmelted parts can be distinguished. An arbitrary demarcation line dividing the melted and nonmelted parts can be defined by choosing an appropriate GAR range. An effective GAR range with distinct boundaries was examined, as shown in Figure 2. The red dotted line denotes the arbitrary demarcation line. It is reported that the boundary between the melted and nonmelted parts can be distinguished to a certain degree [19,20]. Here, the melted and nonmelted parts were tried to be divided by applying a quantitative criterion using EBSD results. When the specific value was 0.35, a large number of white grains near the arbitrary demarcation line were not included in the melted part (Figure 2a). When the specific value was 0.55, a large number of grains from the nonmelted part (matrix in bare wire) were recognized as columnar grains (Figure 2c). However, when the specific value was in the range 0 < GAR < 0.45, the melted part and the nonmelted part were classified effectively, as shown in Figure 2b. From these results, the demarcation line can be defined by referring to the range 0 < GAR < 0.45. Figure 3 shows an example, wherein a demarcation line was drawn on the molten mark according to the established criterion, and it was possible to distinguish the melted and non-melted parts clearly. As a result of applying this method to various molten marks, it is noted as an appropriate method for determining a demarcation line.

### 3.2. Boundary Characteristics Analysis

**Figure 4:**
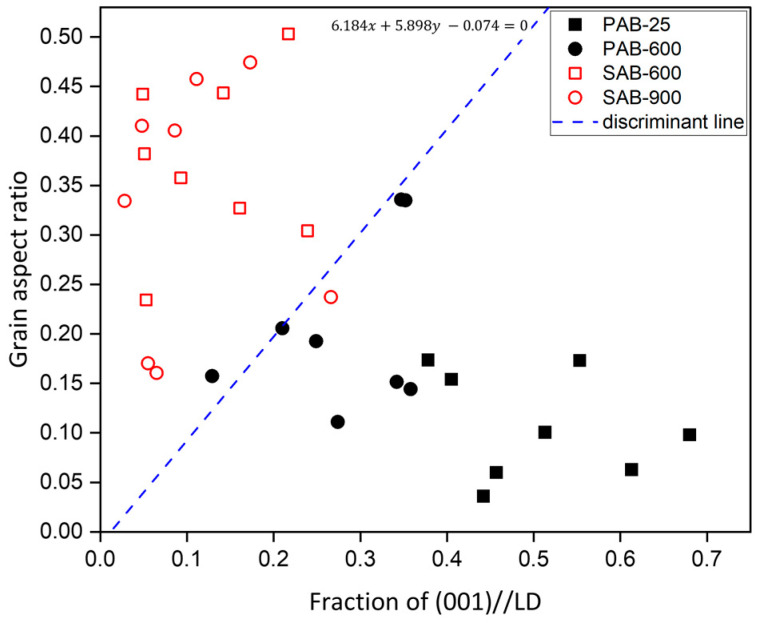
Data distribution according to the grain aspect ratio (GAR) and fraction of (001) perpendicular to demarcation line (//LD) in primary-arc beads (PABs) and secondary-arc beads (SABs).

Figure 4 shows the discriminant result of PABs and SABs using the GAR and the fraction of the (001)-orientation component that is perpendicular to the demarcation line. Here, we defined the perpendicular direction of the demarcation line as a longitudinal direction (LD). The discriminant method is detailed in a previous study [14]. Here, linear-discriminant analysis (LDA) was applied, creating a decision boundary by learning the data distribution and then classifying the data. The relevant equation is:

(1) $${\left(\mu_{1}-\mu_{2}\right)}^{T}\Sigma^{-1}x-[{\left(\mu_{1}-\mu_{2}\right)}^{T}\Sigma^{-1}\left(\mu_{1}+\mu_{2}\right)]/2-\ln\left[P\left(W1\right)/P\left(W2\right)\right]=0$$

where *μ*_1_ and *μ*_2_ are the average value for the GAR and the average value for the fraction of the (001)-orientation component in PABs and SABs, respectively, and *P*(*W*1) and *P*(*W*2) are the prior probabilities of PABs and SABs, respectively. 

In Figure 4, most data can be differentiated correctly using the discriminant method proposed in the previous report [14]. Nevertheless, the two molten marks of PAB-600 and SAB-900 did not satisfy the suggested discriminant criterion. It can be seen that the data corresponding to PAB-600 were distributed near the discriminant line because even after the short circuit, the molten mark is continuously exposed to 600 °C, causing recrystallization, coarse grains, and grain growth with diverse orientations. Consequently, the (001)//LD texture was weakened, and the GAR became larger, resulting in the distribution of PAB-600 around the discriminant line.

As mentioned in the introduction, distinguishing PABs and SABs is limited owing to using only two discriminant factors, grain aspect ratio and the fraction of (001) parallel to LD. Therefore, discriminant factors, in addition to the above parameters, should be considered to achieve high reliability in the discrimination of the molten marks.

**Figure 5:**
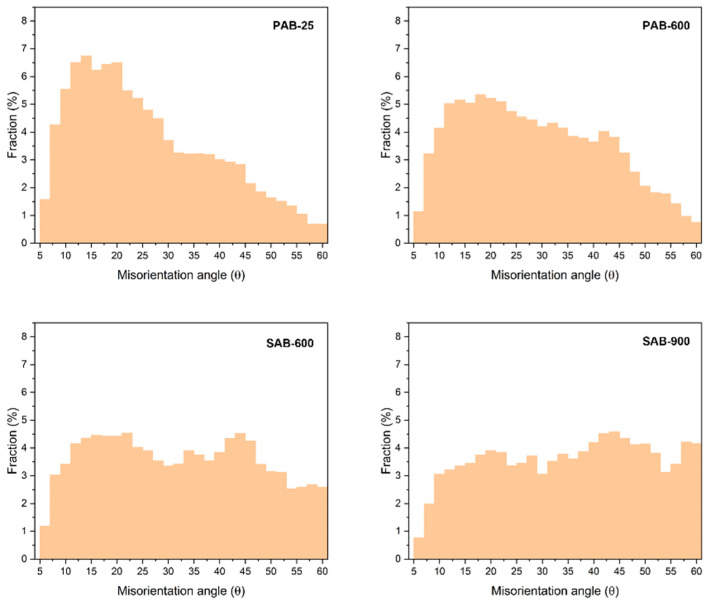
Misorientation distribution of primary-arc beads (PABs) and secondary-arc beads (SABs).

Figure 5 shows the average fractions of PAB-25, PAB-600, SAB-600, and SAB-900 according to the misorientation angle (*θ*). The number of samples of each PAB and SAB is 16. PABs showed relatively high fractions compared to SABs in lower-angle boundaries (*θ* < 40°), whereas SABs showed higher fractions in higher-angle boundaries (*θ* > 40°). 

**Figure 6:**
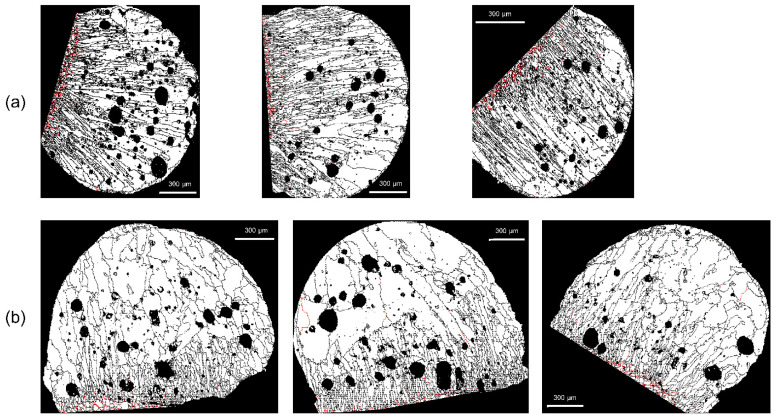
Distribution of Σ3 boundaries (red line) in the molten marks: (**a**) PAB-25 and (**b**) PAB-600.

**Figure 7:**
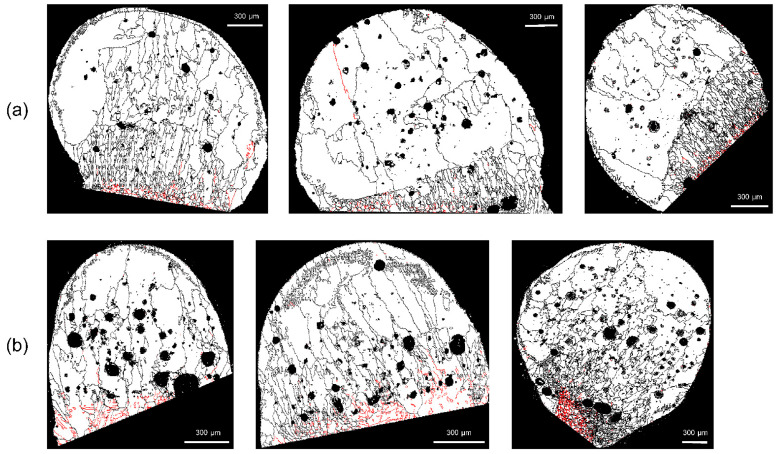
Distribution of Σ3 boundaries (red line) in the molten marks: (**a**) SAB-600 and (**b**) SAB-900.

The relatively high fractions of SABs in the range 55° < *θ* < 60° are noteworthy. The high fraction around *θ* = 60° is related to the recrystallization behavior during heat treatment. The drawn copper wire stores plastic-deformation energy in the form of dislocations. Recrystallization occurs readily when a copper wire is heated owing to a fire. The stored energy by the dislocations is the driving force for recrystallization [21]. During recrystallization, grain-boundary migration is accompanied by forming coherent twin boundaries in face-centered cubic metals, such as copper, nickel, and austenitic steel [22]. A coherent twin boundary is a boundary in a stable state in which the crystal lattices of two grains form an angle of 60° with respect to the (111) plane axis [23], which explains the higher fraction of SABs around *θ* = 60°. This kind of twin boundary is called a Σ3 CSL boundary. The twin-related crystal lattices have one-third of their atomic positions in common, which corresponds to a Σ3 boundary [24]. The unique feature of Σ3 boundaries, developed via heat treatment (a fire), can be useful in distinguishing between PABs and SABs. Figure 6 and Figure 7 show the location of the Σ3 boundaries in PABs and SABs on the EBSD maps. Most of the Σ3 boundaries (red lines) were located around the demarcation line, and Σ3 boundaries were more distributed in SABs.

Table 2 shows the average fraction of Σ3 boundaries in each molten mark. The fraction of Σ3 boundaries in PAB-25 is the smallest at 1.796%, whereas that in SAB-900 is the largest at 9.801%. In general, PABs are short-circuited at room temperature, but reheating occurs because of a fire, so it is necessary to observe the change in Σ3 boundaries of PAB-600. The fraction of Σ3 boundaries in PAB-600 is 2.197%, which is 0.401% larger than that of PAB-25. It is possible that the recrystallization occurred during heating at 600 °C, so Σ3 boundaries developed. The difference in the fraction of Σ3 boundaries between PABs and SABs is remarkable. Notably, the ambient temperature significantly affects the development of Σ3 boundaries around the demarcation line just before the short circuit occurs. This phenomenon can be explained using the results obtained by Baker et al. [25]. They observed that the fraction of Σ3 boundaries increased as the temperature gradient at the front of the hot zone decreased, and the annealing temperature increased during the directional annealing of cold-rolled copper single crystals. Therefore, when SABs are short-circuited, a moderate temperature gradient and a high annealing temperature increase the fraction of Σ3 boundaries around the demarcation line. In contrast, the fraction of Σ3 boundaries in PABs is relatively small compared to SABs because they short-circuit at room temperature and have a high-temperature gradient, so annealing is suppressed. Consequently, the fraction of Σ3 boundaries can be chosen as a discrimination factor.

### 3.3. Grain Size Analysis

The characteristic of grain size was used as a discriminant factor between PABs and SABs. The grain size should be calculated after eliminating errors and artifacts during EBSD measurement. Some errors may remain even after the cleanup during post-processing. Therefore, to measure the grain size, the minimum grain size (*D_m_*) must be determined [26,27]. If grain size (area) is calculated by excluding grains smaller than *D_m_*, the reliability of the measurement results can be increased. In general, to reduce grain size measurement errors, the number of pixels included in one grain should be more than 5 [28]. 

**Figure 8:**
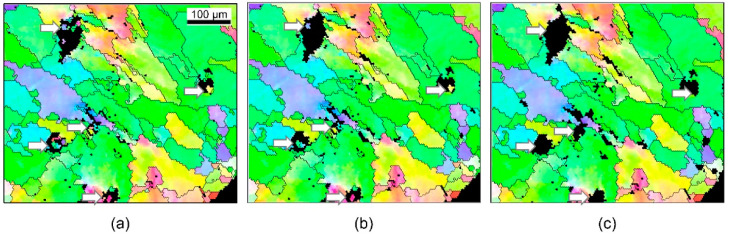
Variations of erroneous grains in voids according to the setup of minimum grain size: number of pixels (**a**) 2, (**b**) 5, and (**c**) 10.

**Figure 9:**
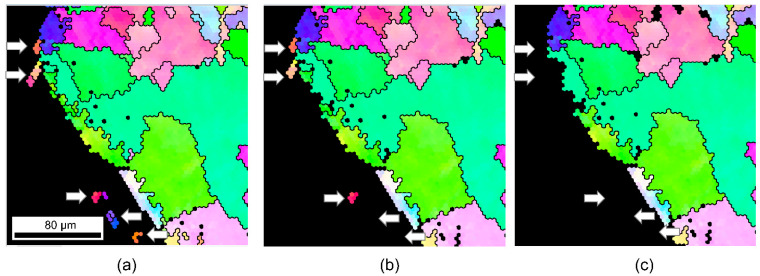
Variations of erroneous grains at points other than the molten mark according to the minimum grain size: pixel sizes of (**a**) 2, (**b**) 5, and (**c**) 10.

With *D_m_* set to 2, 5, or 10 pixels, checks were conducted to determine whether or not the erroneous grains were deleted. As shown in Figure 8, there are many voids in the molten marks, and these voids often result in erroneous grains. As indicated by the white arrows, when *D_m_* is 2 or 5 pixels, errors remain (Figure 8a,b); the errors disappear when *D_m_* is set to 10 pixels. In Figure 9, the arrows indicate the erroneously measured grains in the region outside the molten mark. With *D_m_* = 10 pixels, the erroneously measured grains were removed. In this EBSD analysis, a step size of 4 μm was used, and considering the hexagonal-shape factor (√3/2), 10 pixels equal 138.6 μm^2^. Therefore, to distinguish between PABs and SABs in copper wire with a diameter of 1.6 mm, the minimum grain size should be over 138.6 μm^2^.

In a previous study, Park et al. [14] observed that PABs have small grains at the demarcation line, and columnar grains grew mainly in the (001) component perpendicular to the demarcation line. In SABs, small grains at the demarcation line were similar to those in PABs; inside the molten part of SABs, however, relatively large grains and relatively equiaxed microstructure were observed. Accordingly, it is expected that a criterion for distinguishing PABs and SABs can be presented by comparing the size (area) of the largest grains from each molten mark. The total size of each molten mark is different, and normalization is required to compare the size of each molten mark. The ratio of maximum grain size (area) to the total area of the molten mark was used as a discriminant parameter. Equation (2) was used to calculate the total area of the molten mark.

(2) $$A=\left(\sqrt{3}/2\right)\alpha \beta^{2}$$

Here, *A* is the total area of the molten mark, √3/2 is the shape factor for the hexagonal pixel (shape factor is 1 when a square pixel is used), *α* is the total number of pixels of the molten mark, and *β* is the step size.

Table 3 shows the average fraction of maximum grain size in each molten mark. The fraction of PAB-25 is the smallest at 3.877%, whereas that of SAB-600 is the largest at 17.380%. Compared with PABs, it can be seen that the fraction in SABs was relatively high. SABs, formed under external heat, have a relatively gentle thermal gradient. This results in lower nucleation rate and grain growth under high temperatures of 600 °C and 900 °C. Therefore, larger grains are formed in SABs. Between PAB-25 and PAB-600, the larger grain size of PAB-600 can be explained by the grain growth during heating at 600 °C after a short circuit [29,30]. 

**Figure 10:**
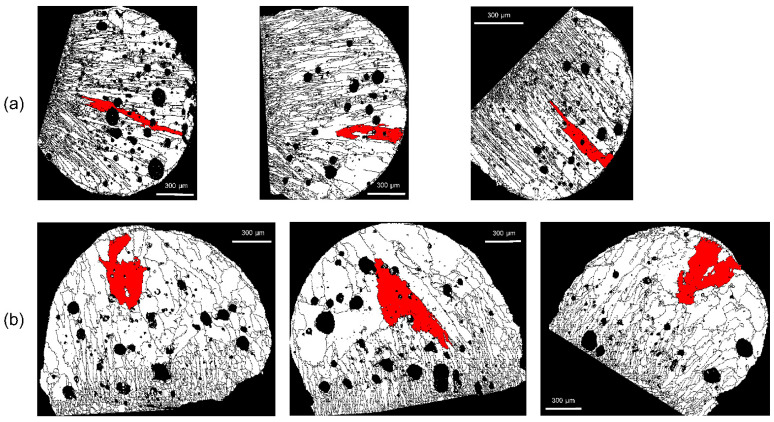
Color representation of the grain with maximum size: (**a**) PAB-25 and (**b**) PAB-600.

**Figure 11:**
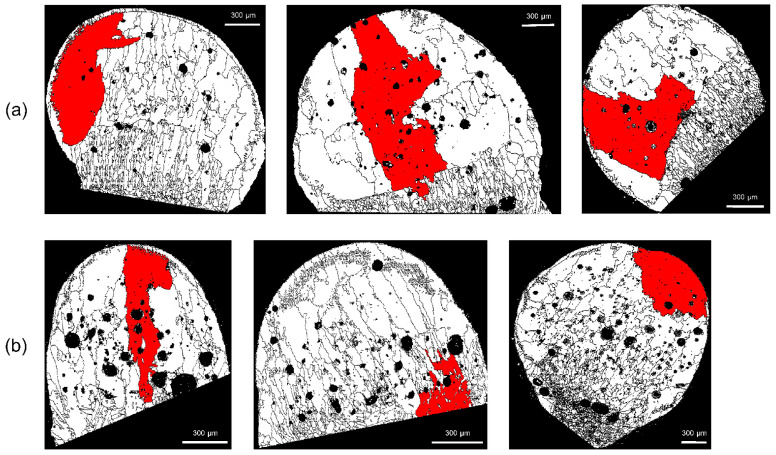
Color representation of the grain with maximum size: (**a**) SAB-600 and (**b**) SAB-900.

Figure 10 and Figure 11 show comparisons of grain sizes of PABs and SABs, by using the grains with maximum grain size (red color). In PAB-25 and PAB-600, the grains are mostly distributed in columnar structures, whereas in SAB-600 and SAB-900, columnar and equiaxed structures are mixed. The maximum grain size of SABs is clearly larger than that of PABs. Considering the observation that SAB-600 has larger grains than SAB-900, it appears that high ambient temperature does not increase the grain size. Thin grains are developed at the outer surfaces of molten marks in both PABs and SABs. The thicknesses of the thin grains in SAB-600 are higher than that in SAB-900. Remarkably, the larger grains developed in SAB-600 are located between thin outer grains and interior columnar grains. Apparent boundaries are visible, which result from large grains adjacent to outer thin grains and columnar grains developed at the demarcation line (Figure 11a). In SAB-900, where the formation of columnar grains is suppressed, visible boundaries are not observed (Figure 11b). The reason for the development of larger grains in SAB-600 is obscure, owing to the complexity of heat transfer, solidification, and nucleation during short-circuit and heating. Three-dimensional microstructure analysis using the focused-ion-beam technique may clarify this mechanism. Accordingly, the fraction of maximum grain size to the total molten-mark area was chosen as a discrimination factor.

### 3.4. Identification of Molten Marks by Classification

**Figure 12:**
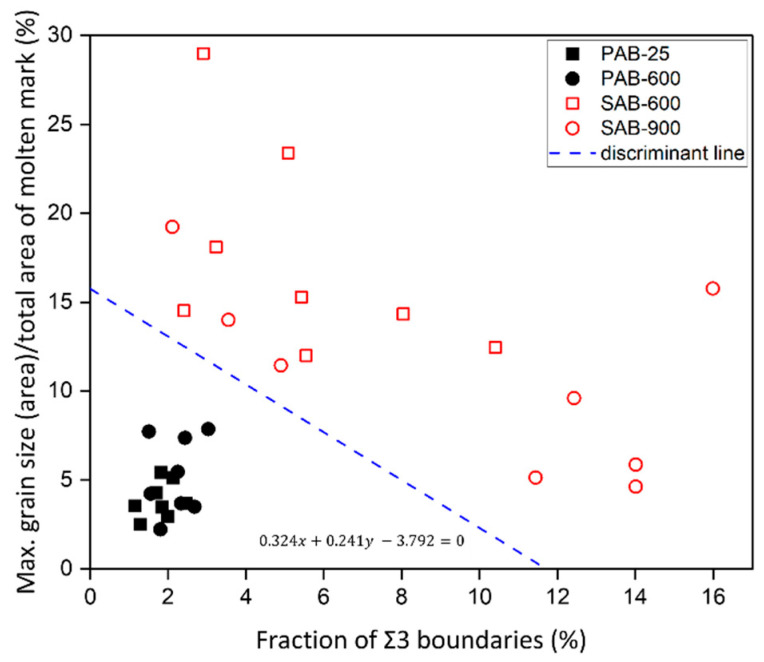
Distribution of primary-arc beads (PABs) and secondary-arc beads (SABs) according to the fraction of Σ3 boundaries and normalized maximum grain size.

Section 3.2 and Section 3.3 evaluated two factors—the fraction of Σ3 boundaries and the ratio of maximum grain size (area) to the total area of the molten mark—to discriminate between PABs and SABs quantitatively. A linear discriminant of equation (1) was used to create a decision boundary to distinguish between PABs and SABs, wherein *μ_1_* and *μ_2_* represent the average value for the fraction of Σ3 boundaries and the average value for the ratio of maximum grain size (area) to the total area of molten mark in PABs and SABs, respectively. A discriminant line (f(x,y) = 0.324x + 0.241y − 3.792) was derived from this equation. Figure 12 represents that the PABs and SABs can be identified based on the new discrimination line. The data corresponding to PABs were distributed in a narrow area with a small deviation, whereas the data corresponding to SABs were located in a wide area with a large deviation. It was estimated that the high ambient temperature in the short circuit resulted in a large deviation, demonstrating the disappearance of unique microstructure characteristics compared to PABs. 

The molten marks were classified using a t-distributed stochastic neighbor embedding (t-SNE) [31], which is a machine-learning algorithm for nonlinear dimensionality reduction with minimum structural-information loss, to check whether PABs and SABs are distinguished when the four discriminant factors are considered simultaneously. 

**Figure 13:**
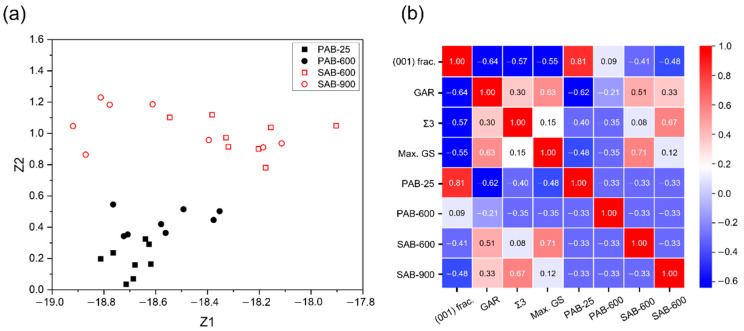
Machine-learning analyses of primary-arc beads (PABs) and secondary-arc beads (SABs): (**a**) t-SNE plot and (**b**) Pearson correlation analysis.

Figure 13a shows that PABs and SABs can be identified using the dimension 2 value; the data corresponding to PABs are located in the area in which the value is lower than 0.6, and the data corresponding to SABs are located in the area in which the value is bigger than 0.6. The molten marks, according to reheating temperature in each PAB and SAB, are relatively indistinguishable; the boundary line between PAB-25 and PAB-600 is somewhat ambiguous, and the data corresponding to SAB-600 and SAB-900 are mixed. As stated above, the t-SNE nonlinearly reduces high-dimensional data to a low-dimensional space, so the axes of the t-SNE plot are difficult to interpret in terms of the original high-dimensional data. For this reason, a Pearson correlation was implemented using the Pandas Python package [32] to identify the effects of the discriminant factors on the type of molten marks. Figure 13b presents the Pearson correlation coefficients between the discriminant factors and the molten marks. The coefficient means the strength of the linear relationship between the two variables. The sign of the coefficient is positive (negative) if the variables are directly (inversely) related. When the coefficient is close to 1 (0), the variables are strongly (weakly) correlated. In Figure 13b, PABs have a negative correlation with the fraction of Σ3 boundaries, the ratio of maximum grain size (area) to the total area of the molten mark, and the GAR, so they have a positive correlation with the (001)//LD. In comparison, SABs have a positive correlation with the fraction of Σ3 boundaries, the ratio of maximum grain size (area) to the total area of the molten mark, and the GAR, and have a negative correlation with the (001)//LD. Therefore, PABs and SABs show opposite correlation tendencies to the discriminant factors, which would cause the separation of PABs and SABs in the t-SNE analysis in Figure 13a. The correlation coefficients of the molten marks according to reheating temperature exhibit the same sign but different values. This would cause the relatively indistinguishable separation of PAB-25 and PAB-600, and SAB-600 and SAB-900 in t-SNE analysis (Figure 13a). Those coefficients in Figure 13b also provide information on the discriminant factor most strongly correlated with the type of molten marks. Regardless of the sign, the factors with the largest coefficient value with PAB-25, PAB-600, SAB-600, and SAB-900 are the (001)//LD, the ratio of maximum grain size (area) to the total area of the molten mark, and the fraction of Σ3 boundaries, respectively. The information obtained from machine-learning analyses would contribute to distinguishing the molten marks.

## 4. Conclusions

In this study, a method that can quantitatively distinguish molten marks using EBSD analysis was investigated. The four discriminant factors, such as the (001)//LD, GAR, fraction of Σ3 boundaries, and fraction of maximum grain size to the total molten-mark area, were verified using the machine-learning analyses, the t-SNE, and Pearson correlation.

Previously suggested discriminant factors [14], such as the (001)//LD and GAR, were limited to specific ambient temperatures after a short circuit. To address these limitations, two new factors, the fraction of the Σ3 boundaries and the ratio of the maximum grain size (area) to the total area of the molten mark, were proposed as discriminant parameters and validated. The quantitative value of the fraction of Σ3 boundaries and the grain-size information was evaluated. SABs had a higher fraction in higher angle boundaries of *θ* > 40° and Σ3 boundaries than PABs. Moreover, the ratio of maximum grain size (area) to the total area of the molten mark was relatively large in SABs. Machine-learning analyses, such as t-SNE and Pearson correlation, provide information on the discriminant factor most strongly correlated with the type of molten marks.

Although this proposed discrimination is limited to bare copper wires with a specific diameter under specific temperatures, the possibility of quantitative discrimination for molten marks using EBSD analysis was confirmed by evaluating distinct factors. In the future, investigating the microstructure characteristics of molten marks in sheathed wires through a compartment fire test is planned.

## Figures and Tables

**Table 1:** The experimental conditions for the fabrication of the molten marks.

Molten Marks	Number of Samples	Heating Temperature(°C)	Heating Time (Minutes)	
			Before Short Circuit	After Short Circuit
PAB-25	8	Ambient	-	-
PAB-600	8	600	-	5
SAB-600	8	600	2	5
SAB-600	8	900	2	5

**Table 2:** Average fraction of Σ3 boundaries in each molten mark.

Molten Mark	PAB-25	PAB-600	SAB-600	SAB-900
Fraction of Σ3 (%)	1.796	2.197	5.378	9.801

**Table 3:** Average fraction of maximum grain size in each molten mark.

Molten Mark	PAB-25	PAB-600	SAB-600	SAB-900
Fraction of maximum grain size (%)	3.877	5.258	17.380	10.240

## Data Availability

The data related to this work can be obtained from the corresponding author upon reasonable request.

## References

[1] Troitzsch J.H. (2016). Fire, statistics, ignition sources, and passive fire protection measures. J. Fire Sci..

[2] Richard C. (2021). Home Fires Caused by Electrical Failure or Malfunction: November 2021.

[3] Liu K.-H., Shih Y.-H., Chen G.-J., Chou J.-M. (2015). Microstructural study on molten marks of fire-causing copper wires. Materials.

[4] National Fire Protection Association (NFPA) NFPA 921: Guide for Fire and Explosion Investigations 2017. https://www.nfpa.org/codes-and-standards/all-codes-and-standards/list-of-codes-and-standards/detail?code=921&year=2017.

[5] Erlandsson R., Strand G. (1985). Investigation of physical characteristics indicating primary or secondary electrical damage. Fire Saf. J..

[6] Ibashi Y., Kishida J. Research on first and second fused mark discrimination of electric wires. Proceedings of the 1990 JAFSE Annual Symposium.

[7] Nobuo M. (1995). Discrimination between primary and secondary arc marks on electric wires by micro-void distribution. Rep. Natl. Police Coll. Res. J..

[8] Miyoshi S. Internal cavity analysis of electrical arc beads. Proceedings of the 4th Asia-Oceania Symposium on Fire Science & Technology, Asia-Oceania Assn. for Fire Science & Technology/Japan Assn, for Fire Science & Engineering.

[9] Lee E.-P., Ohtani H., Seki T., Hasegawa H., Imada S. (2000). Discrimination between primary and secondary molten marks on electric wires by DAS. J. Appl. Fire Sci..

[10] Lee E.-P., Ohtani H., Matsubara Y., Seki T., Hasegawa H., Imada S., Yashiro I. (2002). Study on discrimination between primary and secondary molten marks using carbonized residue. Fire Saf. J..

[11] Satoh K., Sugisaki M., Kakizaki S., Itoh C., Saitoh K., Iwaki M. (1996). Secondary ion mass spectroscopy (SIMS) and auger electron spectroscopy (AES) applied to the fire investigation for short circuit. Proceedings of the 1996 Annual Meeting of Japan Association for Fire Science and Engineering.

[12] Anderson R.N. (1996). Which came first … the arcing or the fire? Review of auger analysis of electrical arc residues. Fire Arson Investig..

[13] Babrauskas V. (2004). Arc beads from fires: Can ‘cause’ beads be distinguished from ‘victim’ beads by physical or chemical testing?. J. Fire Prot. Eng..

[14] Park J., Kang J.-H., Lee E.P., Ko Y.H., Bang S.B. (2021). New approach to distinguish copper molten marks based on quantitative microstructure analysis using electron backscatter diffraction. Fire Technol..

[15] (2019). Standard for Arc-Fault Circuit-Interrupters 2019.

[16] Weinschenk C., Madrzykowski D., Courtney P. (2020). Impact of flashover fire conditions on exposed energized electrical cords/cables. Fire Technol..

[17] Samuels L.E. (2003). Metallographic Polishing by Mechanical Methods.

[18] Kang J.-H., Kim S.-H. (2010). Sample preparation for EBSD analysis: Tips for metals with delicate surfaces. Kor. J. Met. Mater..

[19] Wright S.A., Loud J.D., Blanchard R.A. (2015). Globules and beads: What do they indicate about small-diameter copper conductors that have been through a fire. Fire Technol..

[20] Roby R.J., McAllister J. (2012). Forensic Investigation Techniques for Inspecting Electrical Conductors Involved in Fire.

[21] Burke J.E. (1950). The formation of annealing twins. JOM.

[22] Carpenter H.C.H., Tamura S. (1962). The formation of twinned metallic crystals. Proc. R. Soc. Lond. A.

[23] Brandon D.G. (1966). The structure of high-angle grain boundaries. Acta Metall. Mater..

[24] Bozzolo N., Bernacki M. (2020). Viewpoint on the formation and evolution of annealing twins during thermomechanical processing of FCC metals and alloys. Metall. Mater. Trans. A.

[25] Baker I., Li J. (2002). Directional annealing of cold-rolled copper single crystals. Acta Mater..

[26] Kim S.-H., Kang J.-H., Han S.Z. (2010). Grain size determination of copper film by electron backscatter diffraction. Korean J. Met. Mater..

[27] (2019). Standard Practice for Determining Average Grain Size Using Electron Backscatter Diffraction (EBSD) in Fully Polycrystalline Materials.

[28] Humphreys F.J. (2001). Review grain and subgrain characterization by electron backscatter diffraction. J. Mater. Sci..

[29] Zhang T., Liu F., Wang H.F., Yang G.C. (2010). Grain refinement in highly undercooled solidification of Ni85Cu15 alloy melt: Direct evidence for recrystallization mechanism. Scr. Mater..

[30] Feng L., Gencang Y. (2001). Stress-induced recrystallization mechanism for grain re-finement in highly undercooled superalloy. J. Cryst. Growth.

[31] Van der Maaten L., Hinton G. (2008). Visualizing Data using t-SNE. J. Mach. Learn. Res..

[32] McKinney W. (2011). pandas: A foundational Python library for data analysis and statistics. Python High Perform. Sci. Comput..

